# Efficacy and safety of postoperative adjuvant chemotherapy with oxaliplatin for elderly patients: results from the CCOG-1302 study

**DOI:** 10.1007/s10147-025-02738-w

**Published:** 2025-03-17

**Authors:** Shinichi Umeda, Goro Nakayama, Takayoshi Kishida, Norifumi Hattori, Koki Nakanishi, Haruyoshi Tanaka, Dai Shimizu, Hideki Takami, Masamichi Hayashi, Mitsuro Kanda, Chie Tanaka, Yasuhiro Kodera

**Affiliations:** Department of Gastrointestinal Surgery, Nagoya University Graduate School of Medicine, 65 Tsurumai-cho, Showa-ku, Nagoya, Aichi 466-8550 Japan

**Keywords:** Colorectal cancer, Adjuvant chemotherapy, CAPOX, Elderly patients, Peripheral sensory neuropathy

## Abstract

**Background:**

Postoperative adjuvant chemotherapy using oxaliplatin in addition to 5-FU-based anticancer agents has become the standard treatment for colorectal cancer, however, there is insufficient evidence regarding the efficacy and safety of oxaliplatin combination therapy in the elderly patients. In this study, retrospective analysis of the results from the CCOG-1302 study was performed to confirm them.

**Methods:**

The patients in the CAPOX continuous (8 courses of CAPOX) and intermittent (2 courses of CAPOX + 4 courses of capecitabine + 2 courses of CAPOX) treatment arms in the CCOG-1302 study were divided into two groups, namely, the elderly (≥ 70) and non-elderly (< 70 years) groups. The adverse events, residual peripheral sensory neuropathy (PSN) and prognosis were analyzed.

**Results:**

The incidence of grade 3 or higher hematologic and non-hematologic toxicities in the continuous and intermittent treatment arm were not significantly different between the elderly and non-elderly groups. During the follow-up period, the percentages of grade I or higher PSN residuals were significantly higher among the elderly individuals in the continuous treatment arm at years 2, 3, 4, and 5. On the other hand, PSN decreased over time in the intermittent treatment arm as well as in the elderly and non-elderly patients. The 3-year DFS was not significantly different between the elderly and non-elderly groups in the continuous and intermittent treatment arms.

**Conclusion:**

Oxaliplatin combination chemotherapy can be safely administered to elderly patients. In addition, intermittent administration may be more useful in elderly individuals for the prevention of PSN.

**Supplementary Information:**

The online version contains supplementary material available at 10.1007/s10147-025-02738-w.

## Introduction

The standard treatment for resectable locally advanced colorectal cancer is surgical resection followed by adjuvant chemotherapy. The prognostic value of 5-FU monotherapy was demonstrated in several trials [1, 2], and the oxaliplatin combination therapy became the standard treatment for resectable locally advanced colon cancer, as NSABP C-07 trial and MOSAIC trial demonstrated the efficacy of oxaliplatin in addition to 5-FU monotherapy [3–5]. As for elderly patients, postoperative adjuvant therapy with 5-FU monotherapy in patients aged 70 years or older has shown similar efficacy in reducing recurrence and prolonging survival as that in patients younger than 70 years, according to analyses of randomized controlled trials conducted in the United States and Europe [6–8]. However, there is no consistent evidence regarding the efficacy and safety of oxaliplatin combination chemotherapy in elderly individuals. Several integrative analyses of the additional benefit with oxaliplatin in elderly patients have been reported [9], while others have failed to observe any benefit with oxaliplatin[8, 10]. In addition, there are reports of an increased incidence of PSN in elderly patients treated with oxaliplatin [11, 12]. Although this is not an adjuvant setting, it has been reported that there was no benefit from adding oxaliplatin to 5-FU as a first-line treatment for unresectable colorectal cancer in elderly patients aged 75 years or older [13]. For these reasons, the Japanese Society for Cancer of Colon and Rectum guidelines 2024 state that the use of oxaliplatin combination therapy in elderly patients aged 80 and over should be determined by weighing up the risks and benefits [14].

CCOG-1302 study, a prospective randomized phase II trial of continuous vs. intermittent oxaliplatin administration in postoperative adjuvant chemotherapy for stage II and III colorectal cancer was previously conducted and it showed that intermittent oxaliplatin reduced the residual effects of peripheral sensory neuropathy (PSN) [15]. In this study, the patients in the CCOG1302 study were divided into two groups: elderly patients aged 70 years or older and non-elderly patients aged less than 70 years and the efficacy and safety of adjuvant chemotherapy with oxaliplatin in elderly patients were evaluated.

## Methods

### Study design

The CCOG-1302 study was an open-label, randomized phase II, non-comparative trial. Patients eligible for the CCOG-1302 study had undergone potentially curative surgical resection for histologically proven stage II (T3–4, N0, M0) or stage III (any T, N1–2, M0) colorectal cancer. The patients were randomly assigned to the continuous or intermittent treatment arm after being stratified according to pathologic T status (T1–3 vs. T4), pathologic N status (N0–1 vs. N2–3), and treatment center. The patients in the continuous treatment arm were treated with eight cycles of CAPOX therapy, consisting of an intravenous infusion of 130 mg/m2 oxaliplatin on Day 1, along with oral administration of 1000 mg/m2 capecitabine twice daily on Days 1–14, repeated every 3 weeks. The patients in the intermittent treatment arm were treated with two cycles of CAPOX therapy followed by four cycles of capecitabine monotherapy, consisting of oral administration of 1000 mg/m2 capecitabine twice daily on Days 1–14, repeated every 3 weeks. Two cycles of CAPOX therapy were reintroduced after four cycles of capecitabine monotherapy.

Present study is subgroup analysis of CCOG-1302 study about elderly patients older than 70 years of age. Patients of the intent-to-treat (ITT) population and safety population in the CCOG-1302 study were divided into two groups (elderly patients aged 70 years or older and non-elderly patients aged less than 70 years).

### Assessment

The primary end-point of this study was the rate of any grade PSN lasting for 1 year from start of treatment, and the key secondary end-point of efficacy was disease-free survival (DFS). The other objectives included overall survival (OS), treatment compliance including reinduction rate of oxaliplatin in the intermittent arm and relative dose intensity (RDI) of study drugs. Adverse events were graded according to the National Cancer Institute Common Terminology Criteria for Adverse Events, version 4.0. PSN was assessed with the use of a standardized checklist by physicians and well-trained nurses. Efficacy was analyzed based on the intent-to-treat (ITT) population, which was defined as eligible and assessable patients who underwent randomization. The safety population was defined as all patients receiving 1 dose of either regimen.

### Statistical analysis

Differences in characteristics between elderly and non-elderly patients were analyzed using the χ2 test for categorical variables and the Mann–Whitney U test for continuous variables. The PSN rates were calculated as proportions with Clopper–Pearson exact confidence intervals. The time-to-event variables DFS were analyzed by the Kaplan–Meier method and compared using the log-rank test. A *P* value < 0.05 was considered to indicate statistical significance. Statistical analyses were performed using JMP 15 software (SAS Institute, Inc., Cary, NC, USA).

## Results

### Patient population

In the ITT population of the CCOG-1302 study, 36 of the 100 continuous-treatment patients were in the elderly group and 64 were in the non-elderly group, while 36 of the 100 intermittent treatment patients were in the elderly group and 64 were in the non-elderly group. In the safety population, there were 34 elderly patients and 64 non-elderly patients among the 98 patients in the continuous arm and 36 elderly patients and 62 non-elderly patients among the 98 patients in the intermittent arm (Supplementary Fig. 1).

The cutoff value for tumor diameter was set at 50 mm, which is closest to the median. There were no significant differences in sex, performance status, tumor size, CEA, tumor depth, lymph node metastasis, pathological stage and risk group between the elderly and non-elderly patients in either the continuous or intermittent arms. There were significantly more left-sided tumors in the elderly group in the continuous-treatment arm (*P* = 0.027) (Table 1).

**Table 1:** Clinicopathologic findings

	Continues arm (*N* = 100)			Intermittent arm (*N* = 100)		
Clinicopathological parameters	elderly group (*n* = 36)	Non-elderly group (*n* = 64)	*P*	Elderly group (*n* = 36)	Non-elderly group (*n* = 64)	*P*
Age, years Median (range)	74 (70–79)	61 (41–69)	-	73 (70–79)	62 (32–69)	-
Sex, n (%)						
Male	19 (53)	33 (51)	0.907	16 (44)	35 (55)	0.325
Female	17 (48)	31 (49)		20 (56)	29 (45)	
ECOG performance status, n (%)						
0	35 (97)	62 (97)	0.923	34 (94)	63 (98)	0.274
1	1 (3)	2 (3)		2 (6)	1 (2)	
Tumor location^†^, n (%)						
Right colon	16 (44)	43 (67)	0.027	24 (67)	46 (72)	0.587
Left colon or rectum	20 (56)	21 (33)		12 (33)	18 (28)	
Tumor size, n (%)						
≤ 50 mm	21 (58)	36 (56)	0.872	18 (50)	34 (53)	0.526
> 50 mm	15 (42)	24 (44)		18 (50)	26 (47)	
CEA, n (%)						
≤ 5 ng/ml	29 (81)	51 (80)		26 (72)	51 (80)	
> 5 ng/ml	6 (17)	8 (13)	0.640	9(26)	8 (13)	0.147
Data missing	1 (2)	5 (7)		1 (2)	5 (7)	
Tumor depth, n (%)						
pT1-3	28 (78)	47 (73)	0.628	26 (72)	50 (78)	0.510
pT4	8 (22)	17 (27)		10 (28)	14 (22)	
Nodal metastasis, n (%)						
pN0,1	27 (75)	51 (80)	0.589	26 (72)	50 (78)	0.510
pN2,3	9 (25)	13 (20)		10 (28)	14 (22)	
TNM stage, n (%)						
II	29 (81)	42 (66)	0.107	30 (83)	45 (70)	0.140
III	7 (19)	22 (34)		6 (17)	19 (30)	
Risk group, n (%)						
Low risk (T1-3 and N0-1)	21 (58)	40 (63)	0.682	21 (58)	40 (63)	0.682
High risk (T4 or N2-3)	15 (42)	24 (37)		15 (42)	24 (37)	

^†^ The cecum, ascending colon and transverse colon were classified as the right colon, and the descending colon, sigmoid colon and rectum were classified as the left colon

*ECOG* Eastern Cooperative Oncology Group, *CEA* carcinoembryonic antigen

### Treatment compliance

There were no significant differences between elderly and non-elderly groups in treatment cycles, treatment completion rates, dose modification rate of capecitabine, total delivery dose of oxaliplatin and RDI of oxaliplatin and capecitabine in both continuous arm and intermittent arm. Oxaliplatin reintroduction rates were also not significantly different in two groups. Dose modification rate of oxaliplatin of elderly patient in intermittent arm was significantly lower compared to non-elderly patients (*P* = 0.04). The total delivery dose of oxaliplatin in elderly patients in the continuous arm was significantly lower than in non-elderly patients (*P* = 0.01). Treatment exposure of patients was summarized in Table 2.

**Table 2:** Treatment exposure

	Continues arm (N = 100)			Intermittent arm (N = 100)		
Parameter	Elderly group (n = 36)	Non-elderly group (n = 64)	*P*	Elderly group (n = 36)	Non-elderly group (n = 64)	*P*
Treatment cycle, times						
Median (range)	8 (0–8)	8 (1–8)	0.81	8 (2–8)	8 (0–8)	0.62
Average (SD)	6.7 (2.0)	6.8 (1.9)	0.82	7.7 (1.0)	7.7 (1.0)	0.93
Completion rate, %						
Overall	64.7	67.2	0.80	88.9	91.9	0.62
Reinduction rate, %						
Oxaliplatin	-	-	-	94.4	93.6	0.86
Dose modification rate, %						
Oxaliplatin	52.9	46.9	0.56	2.7	14.5	0.04
Capecitabine	41.8	35.9	0.61	27.8	30.7	0.76
Median total delivery dose						
Oxaliplatin, mg/m2	800	845	0.67	520	520	0.48
Capecitabine, g/body	268	303	0.01	302	336	0.09
Median relative dose intensity, %						
Oxaliplatin	66.3	71.9	0.39	95.1	93.1	0.67
Capecitabine	70.9	73.6	0.67	92.6	91.6	0.68

*SD* standard deviation

### Safety

The incidence of grade 3 or higher hematologic toxicity in the continuous arm was not significantly different between the elderly and non-elderly groups (24% vs. 25%, *P* = 0.872); moreover, the incidence of grade 3 or higher non-hematologic toxicity was not significantly different between the two groups (18% vs. 20%, *P* = 0.102) (Table 3). In the intermittent arm, the incidence of grade 3 or higher hematologic toxicity was not significantly different between the elderly and non-elderly groups (8% vs. 5%, *P* = 0.494), and the incidence of grade 3 or higher non-hematologic toxicity was not significantly different between the two groups (17% vs. 19%, P = 0.739) (Table 4).

**Table 3:** Adverse effects of continuous treatment arm

	All grades				*P*	Above grade 3				*P*
	Elderly group (n = 34)		Non-elderly group (n = 64)			Elderly group (n = 34)		Non-elderly group (n = 64)		
	*n*	%	*n*	%		*n*	%	*n*	%	
Hematologic toxicity	30	88	52	81	0.363	8	24	16	25	0.872
Neutropenia	22	65	42	66	0.928	5	15	11	17	0.750
Thrombocytopenia	24	70	31	48	0.033	2	6	6	9	0.538
Anemia	22	65	31	48	0.122	0	0	0	0	1.000
Non-hematologic toxicity	33	97	63	98	0.654	6	18	13	20	0.102
Anorexia	25	74	44	69	0.620	0	0	4	6	0.061
Nausea/vomiting	17	50	37	58	0.460	17	50	37	58	0.460
Diarrhea	9	26	22	34	0.419	2	6	1	2	0.237
Allergic reaction	3	9	3	5	0.427	1	3	3	5	0.670
PSN	31	91	58	91	0.928	3	9	5	8	0.863
Grade 2	–	–	–	–	–	15	44	23	36	0.430
HFS	20	59	31	48	0.326	0	0	2	3	0.189
Grade 2	–	–	–	–	–	4	12	14	22	0.205

*PSN* peripheral sensory neuropathy, *HFS* hand foot syndrome

**Table 4:** Adverse effects of intermittent treatment arm

	All grades				*P*	Above grade 3				*P*
	Elderly group (*n* = 36)		Non-elderly group (*n* = 62)			Elderly group (*n* = 36)		Non-elderly group (*n* = 62)		
	*n*	%	*n*	%		*n*	%	*n*	%	
Hematologic toxicity	26	72	50	81	0.340	3	8	3	5	0.494
Neutropenia	20	56	34	55	0.945	2	6	3	5	0.877
Thrombocytopenia	17	47	25	40	0.506	1	3	1	2	0.699
Anemia	16	44	28	45	0.945	0	0	0	0	1.000
Non-hematologic toxicity	33	92	62	100	0.013	6	17	12	19	0.739
Anorexia	21	58	36	58	0.979	2	6	2	3	0.581
Nausea/vomiting	16	44	26	42	0.809	2	6	2	3	0.581
Diarrhea	13	36	19	31	0.579	1	3	1	2	0.699
Allergic reaction	1	3	2	3	0.900	0	0	0	0	1.000
PSN	28	78	60	97	0.003	1	3	2	3	0.901
Grade 2	–	–	–	–	–	7	19	9	14	0.528
HFS	17	47	51	82	< 0.001	2	6	7	11	0.327
Grade 2	–	–	–	–	–	9	25	21	34	0.358

*PSN* peripheral sensory neuropathy, *HFS* hand foot syndrome

**Fig. 1:**
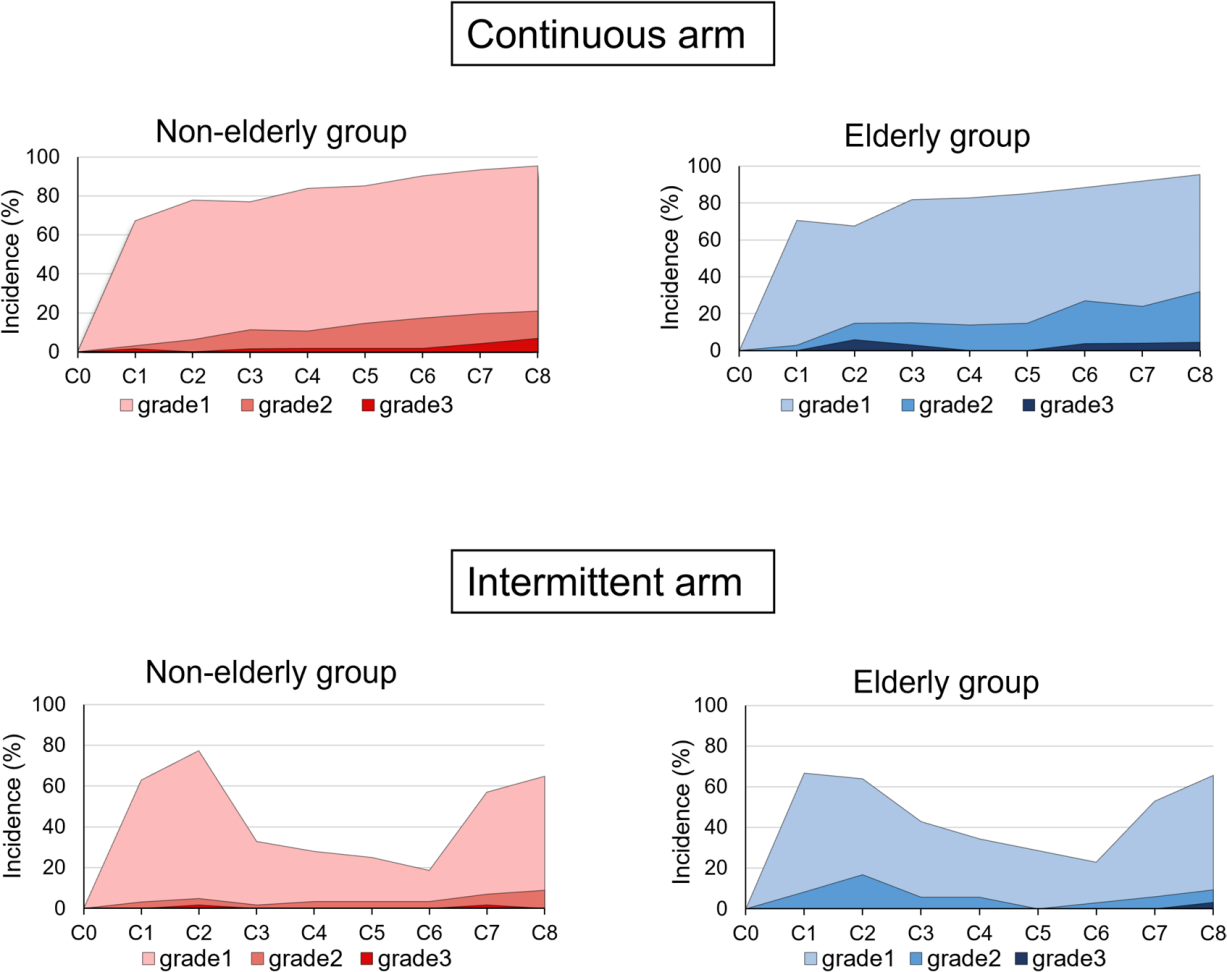
Incidence of peripheral sensory neuropathy during the treatment period

**Fig. 2:**
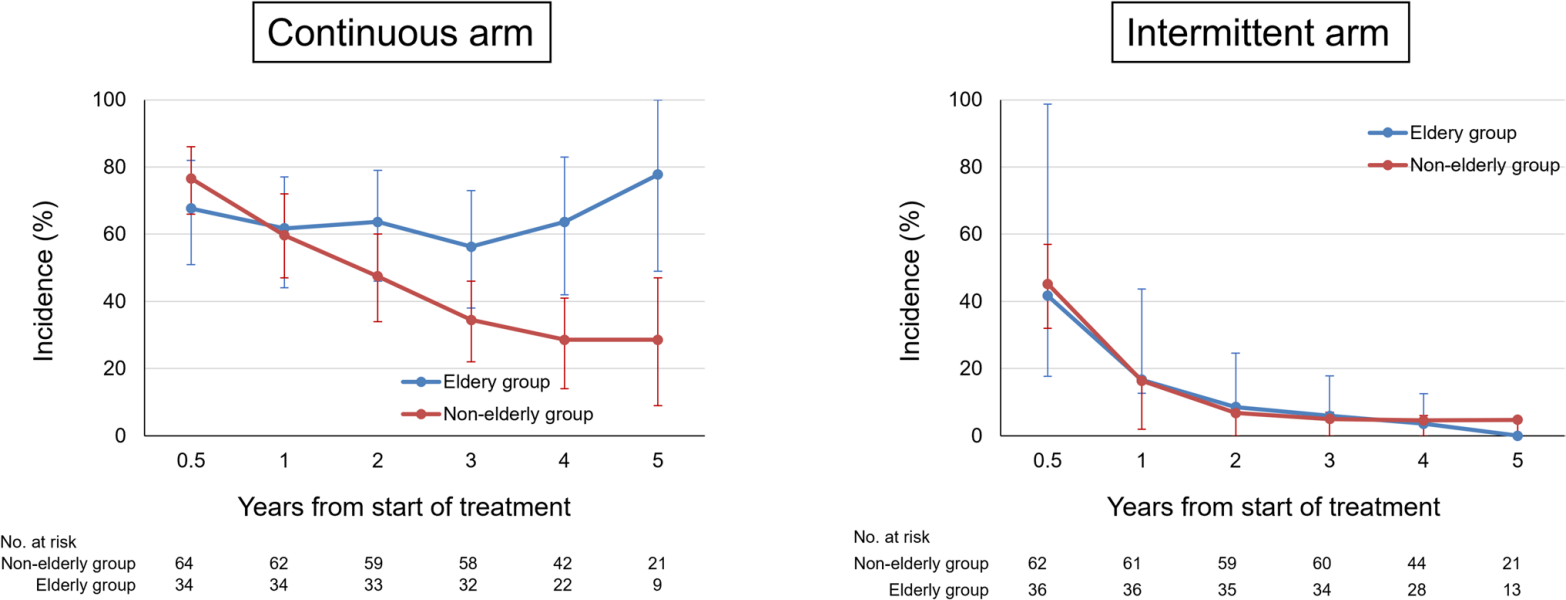
Time-dependent changes in peripheral sensory neuropathy during the follow-up period

The incidence of peripheral neuropathy during the treatment period was similar in the elderly and non-elderly groups in both the continuous and intermittent treatment groups (Fig. 1). The incidence rates of grade 1 or higher peripheral neuropathy at the end of C8 were 95% and 95% in the elderly and non-elderly patients in the continuous arm, respectively, while they were 66% and 65% in the elderly and non-elderly patients in the intermittent arm. During the follow-up period, in continuous-treatment arm, PSN decreased over time in the non-elderly group, while PSN did not improve in the elderly group. The percentages of grade 1 or higher PSN residuals were significantly higher among the elderly patients in the continuous arm at years 2, 3, 4, and 5. On the other hand, in the intermittent arm, PSN decreased over time overall as well as in the elderly and non-elderly patients (Fig. 2).

### Efficacy

**Fig. 3:**
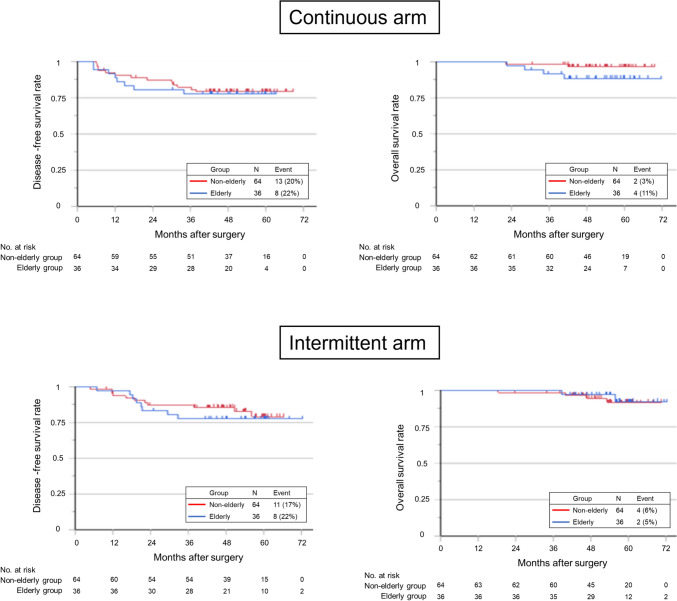
Kaplan–Meier analysis of disease-free survival and overall survival in the elderly and non-elderly groups

Median observation period was 54.0 and 54.2 months in continuous and intermittent treatment arm respectively. There was no significant difference in DFS between the elderly and non-elderly groups in the continuous and intermittent treatment arms, (HR, 1.27; 95%CI 0.51–3.17; *P* = 0.80 and HR, 3.56; 95%CI 0.65–19.4; *P* = 0.14, respectively) (Fig. 3a, b). There was also no significant difference in OS between the elderly and non-elderly groups in the continuous and intermittent treatment arms, (HR, 3.56; 95%CI 0.65–19.4; *P* = 0.14 and HR, 0.78; 95%CI 0.14–4.28; *P* = 0.78, respectively) (Fig. 3c, d). Direct comparison of prognosis between continuous and intermittent treatment arm in elderly patients showed no significant difference in DFS, OS (HR, 0.93; 95%CI 0.40–2.85; *P* = 0.89 and HR, 0.44; 95%CI, 0.08–2.44; *P* = 0.32, respectively) (Supplementary Fig. 2).

## Discussion

In the present study, the continuous and intermittent oxaliplatin arms of the CCOG-1302 trial were analyzed to evaluate the efficacy and safety of oxaliplatin combination chemotherapy in patients aged 70 years or older. No difference was found in efficacy between the two groups; there was no significant difference in grade 3 or higher adverse events, and there were significantly higher rates of residual peripheral neuropathy in the elderly patients in the continuous arm than in the non-elderly patients at 3, 4, and 5 years after adjuvant chemotherapy.

Since there were no significant differences in clinicopathologic factors, such as tumor factors, between the elderly and non-elderly patients in the continuous and intermittent treatment arms, it was thought that a similar population could be compared oncologically to some extent, although no matching was performed. The incidence of grade 3 or higher adverse events was compared between the elderly and non-elderly patients in the continuous and intermittent treatment arms, and the incidence of adverse events was the same in both groups, suggesting that oxaliplatin combination therapy may be safe for elderly patients.

The RDI of oxaliplatin did not differ between the elderly and non-elderly patients in the intermittent arm. On the other hand, the RDI in the continuous arm was 66.3% for the elderly patients and 71.9% for the non-elderly patients, although the difference was not significant, suggesting that the elderly group has more difficulty in continuing oxaliplatin administration. The results also suggest that planned withdrawal of oxaliplatin may allow elderly patients to use the same level of oxaliplatin as non-elderly patients. In fact, the total dose of oxaliplatin in the elderly patients in the intermittent group was 65% of the total dose in the continuous group, because the total dose of oxaliplatin was 800 mg/m^2^ for the continuous administration group and 520 mg/m^2^ for the intermittent administration group. The total dose of capecitabine tended to be significantly higher in the intermittent group because capecitabine was administered systematically during the oxaliplatin withdrawal period [15]. These factors may be related to the fact that the intermittent group had the same level of usefulness as the continuous group.

The incidence of PSN during the treatment period was lower in the intermittent group, and peripheral neuropathy at C8 was also lower in the intermittent group. This trend was similar in the elderly and non-elderly patients. However, follow-up after the completion of chemotherapy revealed that PSN improved over time in both the elderly and non-elderly patients in the intermittent arm, whereas it improved over time in the non-elderly patients in the continuous group but not in elderly individuals, which suggests that continued oxaliplatin administration to elderly patients should be avoided from the perspective of PSN remnants. The reason for few residual PSNs in the intermittent administration group, despite patients received about 65% of the dose of oxaliplatin administered to the continuous group, is thought to be due to intermittent administration. In other words, planned withdrawal of oxaliplatin reduces damage to peripheral nerves.

Regarding the reason for the persistence of PSN in elderly individuals, oxaliplatin-induced PSN is thought to be caused by damage to ganglion cells and nerve fibers due to platinum compounds that accumulate in nerve cells [16]. In the elderly, peripheral nerve aging occurs and they often have peripheral neuropathy due to various diseases that are characteristic of the elderly, therefore it has been suggested that continuous use of oxaliplatin may cause irreversible damage to peripheral nerves [17, 18]. Although there is currently no evidence for the use of drugs to prevent peripheral neuropathy, there is a report that exercise therapy for elderly individuals attenuates PSN, and its introduction may be considered for elderly patients treated with continuous oxaliplatin [19].

Recently, in the IDEA collaboration study, the administration period of oxaliplatin combination therapy as postoperative adjuvant chemotherapy for Stage III colon cancer was analyzed in an integrated analysis of six randomized controlled trials. As a result, in CAPOX therapy, the 4-cycle administration group showed the same recurrence suppression effect as the 8-cycle administration group in patients with a low risk of recurrence. In addition, the 4-cycle administration group reduced adverse events, including peripheral neuropathy [20]. These results led a trend toward reducing the duration of adjuvant therapy for patients at low risk of recurrence. Our results also support the results of the IDEA collaboration. Furthermore, our intermittent oxaliplatin regimen allows continuous capecitabine administration during the oxaliplatin-free period, suggesting that it may be a treatment option for elderly patients with high-risk colorectal cancer.

There are several limitations to this study. Because of the small number of patients in each group, a large phase III study is needed to further clarify the findings and to demonstrate the efficacy and safety of intermittent oxaliplatin in elderly patients. The objective evaluation method is an issue since physician-judged evaluations may not reflect the true feelings of patients and objective method of measuring PSN is needed. The mechanism of residual peripheral neuropathy in elderly patients is unknown, and the development of preventive strategies is awaited.

In conclusion, our results suggest that oxaliplatin combination chemotherapy for colorectal cancer patients can be safely administered to elderly patients. In addition, continuous oxaliplatin administration causes residual PSN, especially in elderly individuals, suggesting that intermittent administration may be more useful in elderly individuals for the prevention of PSN.

## Supplementary Information

Below is the link to the electronic supplementary material.

Supplementary file1 (PNG 299 KB)

Supplementary file2 (PNG 377 KB)

## Data Availability

Due to the nature of this research, participants of this study did not agree for their data to be shared publicly, so supporting data are not available.
