# Supplementary figures and images for: Efficacy and safety of postoperative adjuvant chemotherapy with oxaliplatin for elderly patients: results from the CCOG-1302 study
Source: Int J Clin Oncol. 2025 Mar 17;30(6):1174–82. doi: 10.1007/s10147-025-02738-w (PMC12122561; doi:10.1007/s10147-025-02738-w)

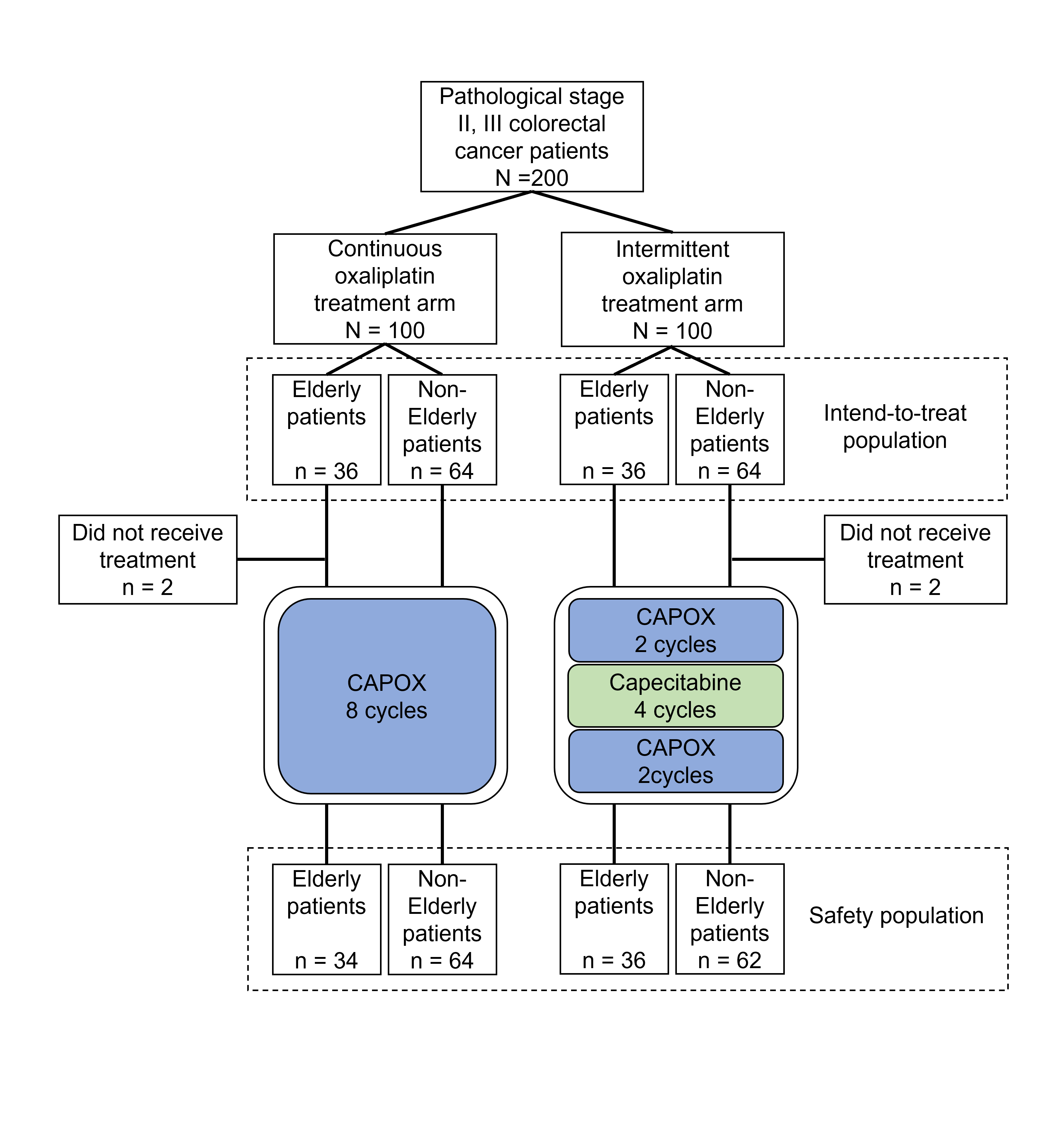

Supplement: Supplementary file 1 — Supplementary file1 (PNG 299 KB) [file 10147_2025_2738_MOESM1_ESM.png]

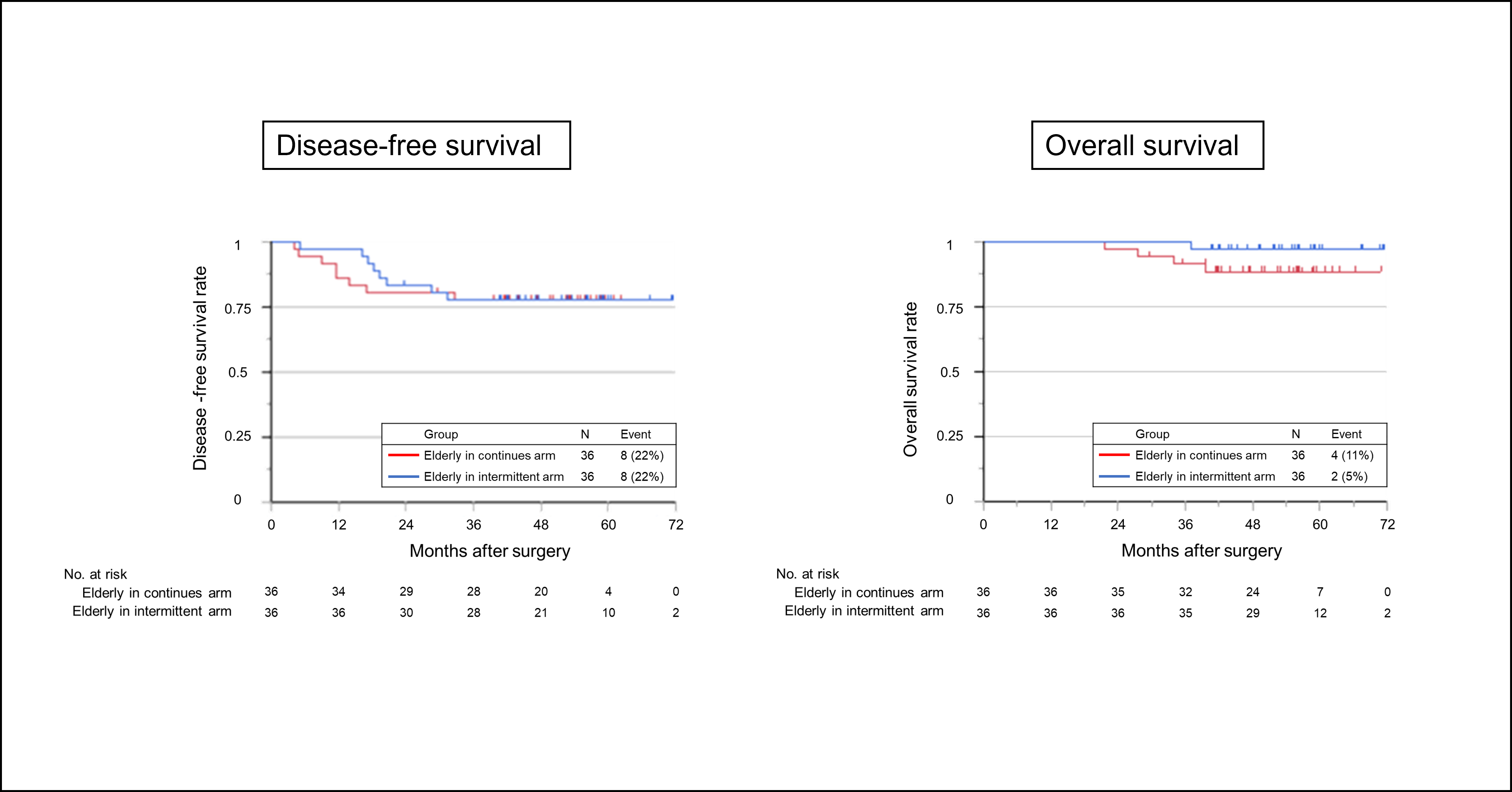

Supplement: Supplementary file 2 — Supplementary file2 (PNG 377 KB) [file 10147_2025_2738_MOESM2_ESM.png]
